# Novel *USH2A* compound heterozygous mutations cause RP/USH2 in a Chinese family

**DOI:** 10.1167/3.9.454

**Published:** 2010-03-17

**Authors:** Xiaowen Liu, Zhaohui Tang, Chang Li, Kangjuan Yang, Guanqi Gan, Zibo Zhang, Jingyu Liu, Fagang Jiang, Qing Wang, Mugen Liu

**Affiliations:** 1 The Union Hospital, Huazhong University of Science and Technology, Wuhan, Hubei, P.R. China; 2 Key Laboratory of Molecular Biophysics of Ministry of Education, College of Life Science and Technology, Center for Human Genome Research, Huazhong University of Science and Technology, Wuhan, China; 3 Departments of Cell Biology and Medical Genetics, Yanbian University, Yanji, China

## Abstract

**Purpose:**

To identify the disease-causing gene in a four-generation Chinese family affected with retinitis pigmentosa (RP).

**Methods:**

Linkage analysis was performed with a panel of microsatellite markers flanking the candidate genetic loci of RP. These loci included 38 known RP genes. The complete coding region and exon-intron boundaries of Usher syndrome 2A (*USH2A*) were sequenced with the proband DNA to screen the disease-causing gene mutation. Restriction fragment length polymorphism (RFLP) analysis and direct DNA sequence analysis were done to demonstrate co-segregation of the *USH2A* mutations with the family disease. One hundred normal controls were used without the mutations.

**Results:**

The disease-causing gene in this Chinese family was linked to the *USH2A* locus on chromosome 1q41. Direct DNA sequence analysis of *USH2A* identified two novel mutations in the patients: one missense mutation p.G1734R in exon 26 and a splice site mutation, IVS32+1G>A, which was found in the donor site of intron 32 of *USH2A*. Neither the p.G1734R nor the IVS32+1G>A mutation was found in the unaffected family members or the 100 normal controls. One patient with a homozygous mutation displayed only RP symptoms until now, while three patients with compound heterozygous mutations in the family of study showed both RP and hearing impairment.

**Conclusions:**

This study identified two novel mutations: p.G1734R and IVS32+1G>A of *USH2A* in a four-generation Chinese RP family. In this study, the heterozygous mutation and the homozygous mutation in *USH2A* may cause Usher syndrome Type II or RP, respectively. These two mutations expand the mutant spectrum of *USH2A*.

## Introduction

Retinitis pigmentosa (RP; OMIM 268000) is characterized by constriction of the visual fields, night blindness, changes of fundi including 'bone corpuscle' lumps of pigment, and loss of central vision. RP is inherited most frequently (84%) as an autosomal recessive trait, followed by autosomal dominant (10%) and has X-linked recessive (6%) patterns in the white USA population. The worldwide prevalence of RP is about 1 in 4,000. RP is the most common hereditary retinal dystrophy causing irreversible blindness. Several loci or genes responsible for RP have been reported.

Retinitis pigmentosa (RP) is a group of hereditary retinal diseases, which are characterized by the degeneration of rod-cone photoreceptors with resultant night blindness and visual field loss. About 20%–30% RP patients have extra-ocular diseases, which are included in more than 30 different syndromes [1]. The most common form of syndromic RP is Usher syndrome, which usually includes both recessive retinitis pigmentosa and hearing loss [2].

On the basis of the severity or progression of the hearing loss, as well as the presence or absence of vestibular dysfunction, Usher syndrome is divided into three clinical subcategories: USH1, USH2, and USH3. Among them, Usher syndrome Type II (USH2) accounts for over 50% of Usher syndrome cases. It is regarded as the most common type, which is characterized by moderate to severe congenital hearing loss, intact vestibular function and postpuberal onset of retinitis pigmentosa [3-5]. Because of mild hearing impairment, USH2 is often misdiagnosed as nonsyndromic RP. To date, three loci have been identified for USH2: *USH2A*, *USH2C* (*GPR98*), *USH2D* (*WHRN*), and the mutations in the *USH2A* gene are responsible for 74%–90% of USH2 cases [6-10].

The *USH2A* gene is located on human chromosome 1q41, and two *USH2A* transcripts were identified: the shorter one consists of 21 exons and the longer one consists of 72 exons [7,11]. In 2004,van Wijk et al. [11] identified additional 51 exons of *USH2A*. The longer isoform of *USH2A* encodes usherin, a transmembrane protein of 5,202 amino acids and contains 68 additional fibronectin repeats [11]. In the inner ears, usherin is transiently expressed in cochlea as an ankle-link complex that connects cochlear hair cells [12,13]. In the retina, usherin is predicted to be a ﬁbrous link that connects the photoreceptor inner segment plasma membrane to the ciliary surface [14,15].

To date, over 80 different mutations in exons 2–21 and 40 mutations in exons 22–72 of the *USH2A* gene have been reported to be associated with Usher syndrome type II, most of which are missense mutations or truncating mutations [7,9,11,16-24]. One of them, c.2299delG, may be the most common mutation among patients because it has been found in 16%–77% of USH2A families [7,18,19,21,23-26]. USH2A mutations have been identified in the patients with a typical USH2 phenotype or nonsyndromic RP [26-29].

In this study, we investigated a four-generation Chinese family with retinitis pigmentosa. After linkage analysis, we mapped the disease-causing gene in the *USH2A* region. Using direct DNA sequence analysis of exons 2–72 and exon-intron boundaries of *USH2A*, we found two novel compound heterozygous mutations: one missense mutation and one splicing site mutation. These two mutations co-segregated with the affected members in the family and were not present in the 100 normal controls. Meanwhile, all members of this family who were initially misdiagnosed as having nonsyndromic RP received an audiometric vestibular test. Results showed that some patients in the family display hearing impairment.

## Methods

### Study subjects and isolation of human genomic DNA

The participants of this study were diagnosed and enrolled at Union Hospital. Informed written consent was obtained from the study subjects. Whole peripheral blood was collected from all participants, and genomic DNA was isolated using the DNA isolation kit for Mammalian Blood (Tiangen Biotech Co., Ltd., Beijing, China). All patients in the family underwent careful ophthalmologic examination, including visual acuity, slit-lamp, fundus ophthalmoscopy, visual field test, and electroretinogram (ERG). Initial RP diagnosis was based on the description of night blindness, typical RP fundus appearance, non-detectable electroretinogram and loss of peripheral visual fields. When the disease gene was mapped to chromosome 1q41 where *USH2A* harbors, patients were given audiometric and vestibular tests. Audiometric tests included otoscopy and standard pure-tone audiometry. Vestibular function was evaluated by caloric test, rotatory chair and electronystagmography. The final clinical diagnosis of USH2 was verified based on typical RP symptoms companied with sensorineural hearing impairment and normal vestibular function.

### Genotyping

A panel of candidate genetic loci for retinitis pigmentosa, including 38 known RP genes, was selected for preliminary linkage and haplotype analysis. The microsatellite markers that flank the 38 known RP genes were selected from the ABI Prism LMS v2.5-MD 10 marker set (Applied Biosystems, Foster City, CA). These markers were genotyped by using an ABI 3100 genetic analyzer (Applied Biosystems). Genotypes were analyzed through GeneMapper 2.5 software (Applied Biosystems).

### Mutation screening

Mutation screening was performed by direct DNA sequence analysis. The complete coding region (exons 2–72) and exon-intron boundaries of *USH2A* were amplified by polymerase chain reaction (PCR). The methods of primers design, PCR amplification, and DNA sequence analysis were performed as previously described [22,23].

### RFLP analysis

Exon 26 of wild type *USH2A* allele contains an NlaIII restriction enzyme site, which the p.G1734R mutation disrupts. We used RFLP analysis to confirm this mutation and test whether the mutation co-segregates with the disease in the family. The 228 bp fragments of exon 26 in *USH2A* gene were amplified from all available family members and the 100 normal controls. The PCR products were digested with 2 units of NlaIII restriction enzyme (New England Biolabs, Inc., Beijing, China) at 37 °C for 5 h. The digested products were separated by a 2.5% agarose gel and visualized by ultraviolet light.

To test if the other novel mutation IVS32+1G>A is the disease-causing mutation, direct DNA sequence analysis was performed for all the family member and 100 normal controls.

## Results

### Clinical examinations

**Figure 1:**
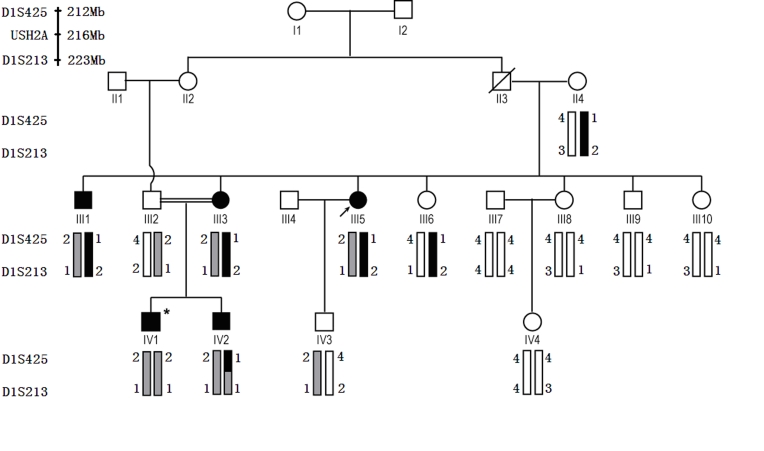
Pedigree structure of the family with USH2/RP. Affected males and females are shown with filled squares and circles, respectively. Normal individuals are shown with empty symbols. The deceased individual is shown with slash. The proband (III:5) is indicated by an arrow. Results from genotyping and haplotype analysis of two markers on chromosome 1 are displayed below each symbol. The disease haplotype is shown with a black or a gray box and the normal haplotypes are shown with open boxes. The *USH2A* gene is located between D1S213 and D1S425. The homozygous individual (IV:1), whose haplotype is different from other patients, is indicated by an asterisk. Four patients (III:1, III:3, III:5) are affected with USH2, and patient (IV:1) displays nonsyndromic RP symptom.

**Figure 2:**
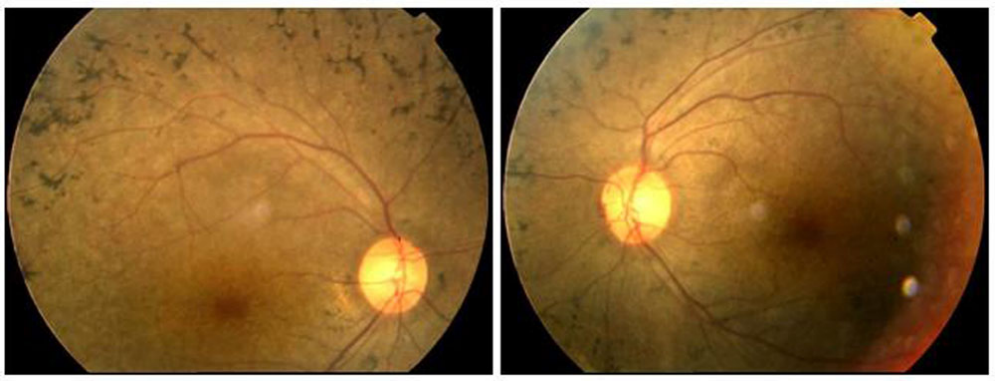
Fundus appearance of the proband. The typical RP symptoms including attenuation of the retinal vessels, waxy pallor of the optic nerve head, and bone speckle-like pigmentation clumps in the peripheral retina were showed.

**Figure 3:**
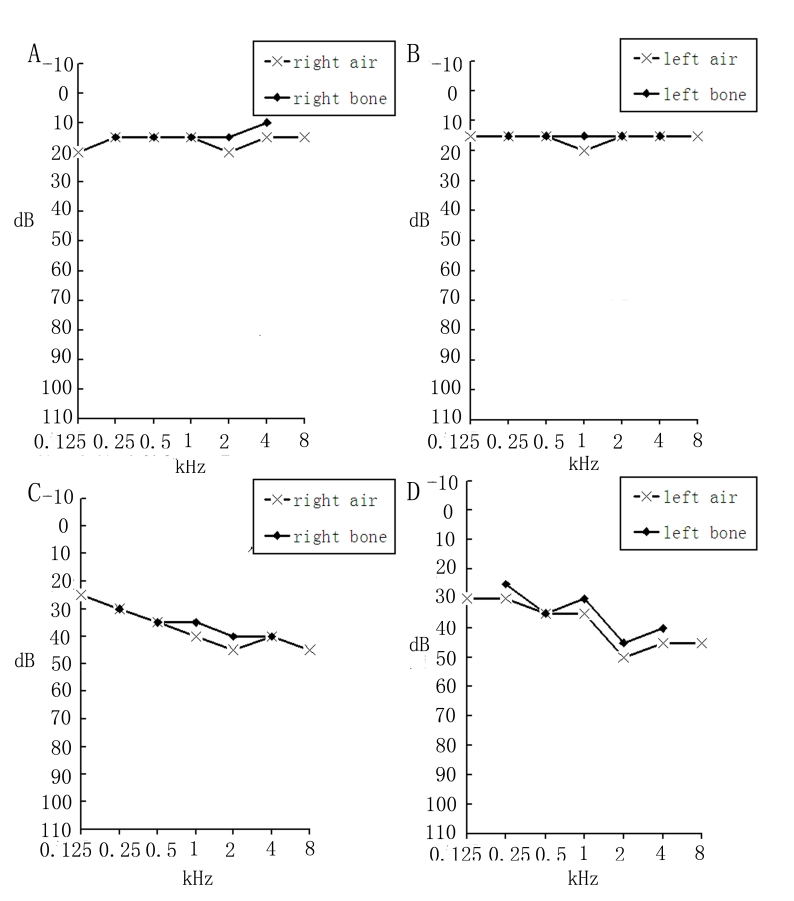
Audiometry results for III:5 and IV:1. **A** and **B** show the result for IV:1 who is normal, **C** and **D** show mild hearing loss for III:5.

Five individuals of the primary study family were having RP or USH2 (Figure 1). The proband (III:5) was a 47-year-old female who experienced night blindness at the age of 23 years as her initial symptoms of RP, which was followed by progressive loss of visual acuity. Her best corrected visual acuity was decreased to finger count level. Fundus examination showed attenuation of the retinal vessels, waxy pallor of the optic nerve head, and bone speckle-like pigmentation clumps in her peripheral retina (Figure 2). The ERG wave amplitudes were unrecordable under scotopic and photopic conditions in both eyes. Similar ophthalmologic examination results were detected in the other two affected siblings (III:1, III:3) of the proband, but the symptom of RP in IV:1 and IV:2 were mild. Audiometric tests of the proband and her siblings indicated mild sensorineural hearing impairment and normal vestibular function while patient IV:1 was normal (Figure 3). His younger brother (IV:2) did not undergo the audiometric test. Detail clinical examination results are shown in Table 1.

**Table 1:** Clinical features of the patients from the Chinese family with Usher syndrome type II.

**Patient number**	**Age (year)**	**Best corrected visual activity (R/L)**	**Fundus appearance**	**Hearing impairment**	**ERG**	**Vestibular function**
III:1	55	FC/FC	RP	Mild	extinct	normal
III:3	53	FC/HM	RP	Mild	extinct	normal
III:5	47	FC/FC	RP	Mild	extinct	normal
IV:1	25	0.15/0.1	RP	Normal	N/A	normal
IV:2	19	0.6/0.6	RP	N/A	N/A	normal

Abbreviations: R represents right eye; L represents left eye; FC represents finger counting; HM represents hand motion; N/A represents data not available.

### Linkage analysis

Genetic linkage analysis excluded all previously identified RP genes except for *USH2A*, which was located between D1S213 and D1S425. Further haplotype analysis suggested that *USH2A* may be the disease-causing gene of this family (Figure 1).

### Mutation analysis

**Figure 4:**
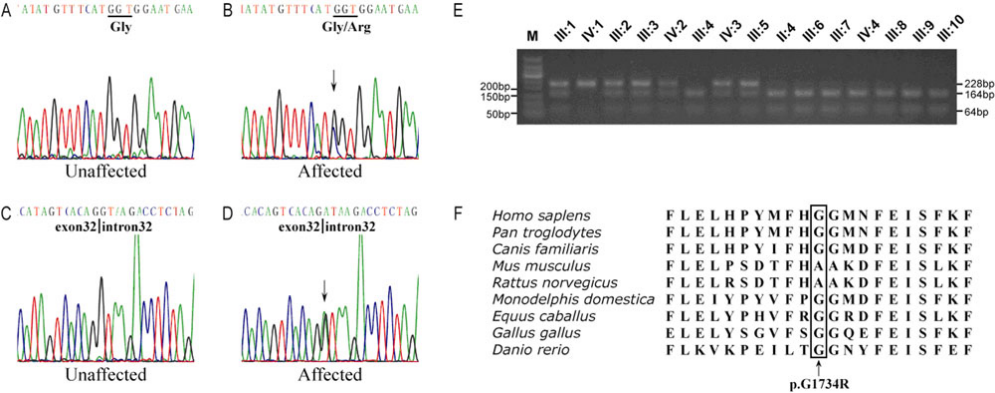
Identification of two novel *USH2A* mutations. DNA sequence analysis for patient III1 showed the presence of compound heterozygous p.G1734R (c.5200G>C) and c.IVS32+1A>T mutations. **A** and **B** show the sequences of a normal and affected family member with mutation p.G1734R (c.5200G>C) allele, respectively. **C** and **D** show the sequences of a normal and affected family member with mutation c.IVS32+1A>T allele, respectively. **E**: Restriction fragment length analysis on the p.G1734R (c.5200G>C) mutation in this study. All the affected individuals (III:1, III:3, III:5, IV:1, IV:2) and the carriers (III:2, IV:3) have three bands (228 bp, 164 bp, and 64 bp), while the unaffected individuals only have two bands (164 bp and 64 bp).The patient IV1 who is homozygous for the p.G1734R (c.5200G>C) mutation displayed only one 228 bp band. **F**: Alignment of the amino acid sequences of laminin G-like domain in the long usherin isoform from different species. Gly1734 (G1734) is conserved during evolution. The box indicates this mutated residue in USH2A.

Direct DNA sequence analysis was performed to identify the *USH2A* gene mutation that caused RP in the family. The complete coding region (exons 2–72) and the exon-intron boundaries of the *USH2A* gene were amplified with the proband DNA and then sequenced. Two novel mutations were found (Figure 4A-D): One was a p.G1734R mutation in exon 26, which resulted in a substitution of glycine for arginine at codon 1734 (p.G1734R). RFLP analysis at this site showed that this mutation co-segregated with the disease in the family (Figure 4E) and was not present in the 100 normal control individuals. The other mutation was a change from G→A at a 5′ splicing site in intron 32(IVS32+1G>A). Direct DNA sequence analysis of each member of the family showed that the splice site mutation was present in all patients except patient IV:1 and was absent in the 100 normal control individuals. All affected members had the compound heterozygote (p.G1734R and IVS32+1G>A) mutation, except for patient IV:1, who is homozygote for the p.G1734R mutation (Figure 4F).

In addition to the two novel pathogenic mutations detected in this study, seven nonpathogenic sequence variants were identified. One of them, c.1935A>T, was novel. The synonymous variant did not cause an amino acid substitution.

## Discussion

In the present study, two novel mutations, p.G1734R and IVS32+1G>A, were identified in a Chinese RP/USH2 family. All the affected individuals were compound heterozygote for p.G1734R and IVS32+1G>A except for patient IV:1, who was homozygous for the p.G1734R mutation because his parents were consanguineous. These results suggest that the compound heterozygous/homozygous mutations in *USH2A* may be the disease-causing mutations in this family.

*USH2A* has been described as the most common locus for syndromic RP [10]. Its mutations have been identified in Colombian, Spanish, Israeli, Canadian, Dutch, and British families with Usher syndrome [21,23,25,30-32]. However, most of the mutations have been restricted to exons 1–21. To date, only one study reported mutation analysis of the complete coding sequence of *USH2A* in the Chinese population, and five mutations (p.R34fs, p.S2828fs, Exon43DEL, p.W3150X, p.T3936P) were identified, four of which were specific to the longer isoform of USH2A [22]. Our study is the second report on this subject in the Chinese population. Both of the novel mutations are located in exons 22–72. Both of these studies suggest that the function of the C-terminal of usherin is important in the retina, and mutations in this region may be responsible for most USH2 cases.

It should be noted that the degree of hearing loss was slightly progressive. Many members in the family-study were unaware that they had partial hearing loss. Furthermore, they were initially misdiagnosed as nonsyndromic retinitis pigmentosa.

We conducted linkage analysis to locate the disease gene and then sequenced the *USH2A* gene. Our results demonstrate that the combination of two mutations affecting the long usherin isoform have a relatively mild effect on audition. Interestingly, three patients who were compound heterozygotes for the two mutations showed Usher syndrome phenotypes, while patient IV:1 was homozygous for the p.G1734R mutation. Upon clinical examination and pure tone audiometry, IV:1 was found to have hearing acuity within the normal range, which may suggest that homozygosity for p.G1734R does not cause hearing loss (Figure 3). Our result is consistent with the findings of Seyedahmadi et al. [24], Rivolta et al. [29], and Kaiserman et al. [33]. Their studies indicated that mutations in *USH2A* may cause retinitis pigmentosa without hearing loss [24,29,33]. However, because IV:1 is 25 years of age, it is possible that he will suffer from hearing impairment in his late years.

**Figure 5:**
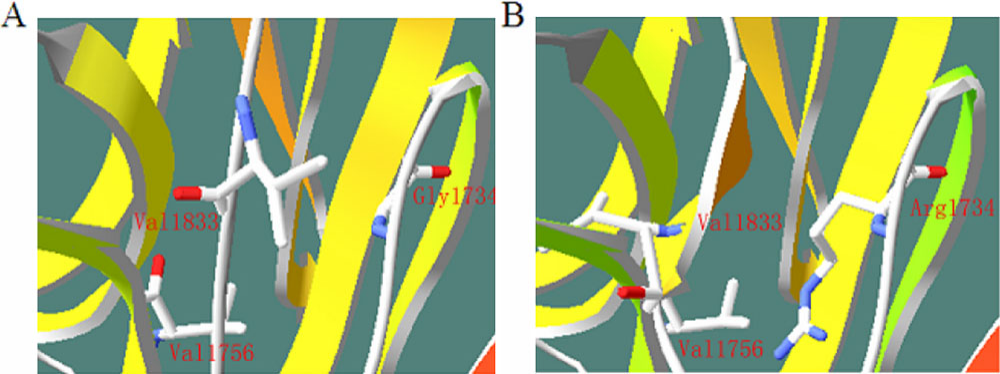
3D modeling of p.G1734R (PDB template 2JD4_B, 26% identity with Lama1). **A**: wild-type protein, **B**: mutant protein. The replacement of Gly by Arg may induce a new secondary structure that includes Leu1831, Val1832, and Val1833. The new secondary structure makes Val1833 very close to Val1756, which may change the conformation of the binding site. The structure of the wild type and the mutant USH2A protein were predicted using Swiss Pdb-Viewer 4.0.1.

The Gly1734 residue is located in the laminin G-like domain (LNS) of USH2A and is highly conserved during evolution. When compared with wild type usherin, the missense mutation of p.G1734R results in the conversion of a non-polar hydrophobic amino acid (glycine) to a positively charged amino acid (arginine). This change might affect the structure and/or function of USH2A and influence the laminin G-like domain of the protein. The LNS domains are usually Ca^2+^ mediated receptors [34]. The substitution of the G1734 of USH2A involved in these structures might result in an abnormal folding of the LNG domain and affect the properties of the protein. We analyzed the structure effect caused by the p.G1734R mutation in USH2A using online Mutagenesis and visualization, which were performed with Swiss Pdb-Viewer 4.0.1 [35-37]. The replacement of Gly by Arg may induce a new secondary structure that includes Leu1831, Val1832, and Val1833. The new secondary structure makes Val1833 similar to Val1756, and may change the conformation of the binding site (Figure 5). Future functional studies may verify the pathogenicity of this missense mutation.

Because of the lack of available tissues from patients, the consequence of the IVS32+1G>A mutation remains unknown. However, IVS32+1G>A mutation is located 5′ donor splice site of intron 32. The splicing mutation maybe skip the exon 32, which contains 162 nucleotides, and lead to a deletion of 54 amino acid residues in USH2A. It is also possible that this mutation could generate an abnormal donor site and result in the disruption of the function of the USH2A protein.

Since van Wijk et al. [11] identified 51 additional exons at the 3′ end of *USH2A* in 2004, the controversy remains whether two alleles with mutations in exons 22–73 could lead to Usher syndrome. Our results provide evidence that these two mutations in the longer isoform can cause Usher syndrome. The compound heterozygotes have a mild effect on audition and the homozygotes for p.G1734R do not cause hearing loss. These findings expand the spectrum of mutations in longer isoform of USH2A and provide useful information for genetic counseling for patients and families with USH2. Further studies may identify the molecular mechanism of the two mutations in the cochlea and the retina.
